# *Arabidopsis* MDN1 Is Involved in the Establishment of a Normal Seed Proteome and Seed Germination

**DOI:** 10.3389/fpls.2019.01118

**Published:** 2019-09-10

**Authors:** Peng-Cheng Li, Jun-Jie Ma, Xi-Meng Zhou, Guang-Hui Li, Chuan-Zhi Zhao, Han Xia, Shou-Jin Fan, Xing-Jun Wang

**Affiliations:** ^1^Biotechnology Research Center, Shandong Academy of Agricultural Sciences, Shandong Provincial Key Laboratory of Crop Genetic Improvement, Ecology and Physiology, Jinan, China; ^2^College of Life Science, Shandong University, Qingdao, China; ^3^College of Life Sciences, Shandong Normal University, Jinan, China

**Keywords:** MDN1, ABI5, seed storage proteins, germination, seed proteome profiling, *Arabidopsis*

## Abstract

Seed germination and formation are the beginning and ending, respectively, of a plant life cycle. These two processes are under fine regulation by the internal genetic information. Previously, we demonstrated that *Arabidopsis* MIDASIN 1 (MDN1) is required for ribosome biogenesis, and its dysfunction leads to pleiotropic developmental phenotypes, including impaired embryogenesis and slow seed germination. In this study, we further found that the weak mutant of *MDN1*, *mdn1-1*, exhibits an increased seed size phenotype. Seed proteomic analysis reveals that a number of proteins involved in seed development and response to external environments are mis-regulated by the MDN1 dysfunction. Many 2S seed storage proteins (SSPs) and late embryogenesis abundant (LEA) proteins are over-accumulated in the dry seeds of *mdn1-1*. Further, some genes encoding seed storage reserves are also upregulated in *mdn1-1* seedlings. More interestingly, abscisic acid-insensitive 5 (ABI5) is over-accumulated in *mdn1-1* seeds, and the loss of its function partially rescues the low seed germination rate of *mdn1-1*. Together, this study further demonstrates that MDN1 is essential for establishing a normal seed proteome, and its mutation triggers ABI5-mediated repression of seed germination.

## Introduction

The seed is an important organ for plant species dispersion (Rodriguez-Gacio Mdel et al., 2009). Seeds are also the most basic and key material in agricultural production because they not only are the main source of starchy food and vegetable oil but also provide people with rich protein.

Seed formation starts with embryo morphogenesis, undergoes a seed maturation process, and ends with seed desiccation (Kroj et al., 2003). During seed maturation, the storage compounds required for germination, including lipids, carbohydrates, and proteins, accumulate in the embryo, the endosperm, or the aleurone layer depending on the species. At this stage, the embryo becomes quiescent and desiccation-tolerant. A dry seed confers the ability to pause the plant life cycle (Bentsink and Koornneef, 2008).

Proteins and lipids are the major substances present in dry seeds of *Arabidopsis*. In particular, 2S and 12S SSPs account for approximately one-third of the seed weight. SSPs function as the main source of nitrogen, carbon, and sulfur for germination and seedling establishment before photoautotrophy. The expression of genes encoding SSPs typically starts in the torpedo-embryo stage and is detected in dry seeds, and their transcriptional regulation is dependent on several B3-domain and basic leucine zipper (bZIP) factors (Kroj et al., 2003). At the later maturation stage, LEA proteins are synthesized. LEA proteins are predicted to be molecular chaperones and prevent protein aggregation under stress conditions, especially drought (Hundertmark and Hincha, 2008). As a result, LEA proteins may be essential for the desiccation tolerance of seeds.

To optimally adapt to various environments, plant seeds time their germination through dormancy (Nee et al., 2017). Under appropriate environment conditions, a dormant seed is wakened and begins germination and seedling establishment. These processes are predominantly mediated by phytohormones abscisic acid (ABA) and gibberellins (GA) (Finkelstein et al., 2008; Nonogaki, 2019).

ABA that is synthesized in embryo and/or endosperm functions in seed maturation and the induction and maintenance of seed dormancy (Kanno et al., 2010). ABA deficiency during seed maturation leads to the absence of seed dormancy (Graeber et al., 2012). In contrast, overexpression of the ABA synthesis genes confers hyperdormancy upon seeds (Nonogaki and Nonogaki, 2017). Through the identification of mutants with altered ABA sensitivity, a number of factors involved in ABA signaling have been identified, including abscisic acid-insensitive 3 (ABI3) and ABI5 (Finkelstein et al., 2002). ABI3 is a B3-domain transcription factor involved in seed maturation (Kroj et al., 2003), and its gene is specifically expressed in developing and germinating seeds (Baumbusch et al., 2004; Bassel et al., 2006). ABI3 acts upstream of ABI5 and is essential for ABI5 expression (Lopez-Molina et al., 2002). ABI5 is a bZIP transcription factor, and its gene expression can be detected in various tissues with strong expression noted in seeds (Albertos et al., 2015). ABI5 plays a role in germination and seedling growth arrest under inappropriate conditions (Lopez-Molina et al., 2001). Furthermore, ABI5 is required for the expression of some *LEA* protein genes (Finkelstein and Lynch, 2000), and the *abi5* mutant displays a large-seed phenotype (Cheng et al., 2014), suggesting that ABI5 is also involved in seed maturation.

In contrast to ABA, GA is involved in promoting seed germination and seedling growth. The balance between these two antagonistic phytohormones is vital for the equilibrium between seed dormancy and germination (Nee et al., 2017). Interestingly, a recent study reported that GA is also required for late embryogenesis, suggesting a synergistic effect on seed maturation with ABA (Hu et al., 2018).

Seed germination is indicated by radicle protrusion, and this process is hypersensitive to a defect in *de novo* protein synthesis (Rajjou et al., 2004). Given that protein synthesis is ribosome-dependent, the conclusion is further suggested by the low germination rate of mutants with a defect in ribosome biogenesis under normal growth conditions (Abbasi et al., 2010; Li et al., 2016; Maekawa et al., 2018). *mdn1-1* (also called *dwarf and short root 1*) is a weak mutant of the *Arabidopsis midasin 1* (*MDN1*) gene resulting from a site-changed mutation (Li et al., 2016). Our previous studies reported that MDN1 is involved in nuclear export of the pre-60S ribosomal particle (Li et al., 2019), and its mutation leads to pleiotropic development phenotypes (Li et al., 2016). In this study, we further found that *mdn1-1* has larger seeds than wild type, and the low germination rate of its seeds is mediated by ABI5 to some extent.

## Materials and Methods

### Plant Materials and Growth Conditions

The wild type used in this study is *Arabidopsis thaliana* ecotype Col-0. The *mdn1-1*, *mdn1-2* (Salk_057010), and *mdn1-1 mdn1-2* (also called *dsr1 mdn1*, bi-allelic mutations in *MDN1*) mutants were described in our previous studies (Li et al., 2016; Li et al., 2019). The relative expression levels of *MDN1* in *mdn1-1*, heterozygous *mdn1-2*, and *mdn1-1 mdn1-2* are reported in **Supplementary Figure 1**. The *abi5* mutant (Salk_013163C) was obtained from Arabidopsis Biological Resource Center. For generation of the double mutant *abi5 mdn1-1*, *abi5* was crossed with *mdn1-1*. Homozygosity of *mdn1-1* was detected by sequencing, and that of *abi5* was verified using PCR amplification of genomic DNA. For seed germination and seedling growth under aseptic conditions, seeds were surface sterilized successively using 70% ethanol (v/v) and 10% H_2_O_2_ (v/v) and then plated on half-strength Murashige and Skoog (MS) medium containing 1% (w/v) sucrose and 0.8% agar. After 3-day stratification at 4°C, plates were moved to a growth chamber with long-day conditions (16 h light/8 h dark) at 22°C. Seven days after germination, plants were transferred into soil.

### Protein Extraction From Mature Seeds of *Arabidopsis* and iTRAQ Analysis

The protein extraction was performed as described in (Wang et al., 2007; Jiang et al., 2017) with some modification. The collected dry seeds were ground to fine powder in liquid nitrogen. The powder was solubilized with BPP extraction buffer (1:3, w/v) of 100 mM Tris-HCl (pH 8.0) containing 100 mM EDTA, 50 mM borax, 50 mM vitamin C, 1% PVPP w/v, 1% Triton X-100 v/v, 2% β-mercaptoethanol v/v, and 30% sucrose w/v. The homogenate was mixed with Tris-saturated phenol (1:1, v/v) and vortexed for 10 min at room temperature. After centrifugation at 15,000*g* for 15 min at 4°C, the supernatant was mixed with BPP extraction buffer (1:1, v/v), and then vortex for 5 min at room temperature. After centrifugation at 15,000*g* for 15 min at 4°C, the supernatant was incubated with five-fold volume of AM precipitator (6.605 g ammonium sulfate in 500 ml methanol) overnight at −20°C. After centrifugation at 15,000*g* for 15 min at 4°C, the supernatant was discarded. The protein pellet was mixed with 1 ml pre-cold methanol and then centrifuged at 15,000*g* for 5 min at 4°C. The protein pellet was mixed with 1 ml pre-cold acetone and then centrifuged at 12,000*g* for 5 min at 4°C (repeat once). After vacuum drying, the pellet was solubilized with lysis buffer (7 M urea, 2 M thiourea, 4% CHAPS, 2% Bio-lyte (pH 3∼10), 1 mM PMSF, and 1% DTT; w/v = 10:1) at 4°C overnight. Following centrifugation at 15,000*g* for 30 min at 4°C, the supernatant was used for SDS-PAGE analysis. The protein concentration was determined using a Pierce™ BCA Protein Assay Kit (P23225, Thermo Fisher Scientific, USA). For SDS-PAGE analysis, three biological replicates of the seed protein extraction were performed. The isobaric tags for relative and absolute quantitation (iTRAQ) experiment was performed by Beijing BangFei Bioscience (China) following the standard protocol of the iTRAQ Reagent-8plex Multiplex Kit (Applied Biosystems, USA). The three biological replicates of wild type were tagged with the 113-, 114-, and 115-Da reporters, while those of *mdn1-1* were tagged with 116, 117, and 118. Liquid chromatography and tandem mass spectrometry (LC-MS/MS) data were extracted and analyzed by the bioinformatics service of Beijing BangFei Bioscience. Peptide and protein identification was performed using “Proteome Discoverer” (Thermo Fisher Scientific, USA) software according to the following parameters: database, uniport—*Arabidopsis thaliana*; enzyme, trypsin; max missed cleavages, 2; fixed modifications, carbamidomethyl (C), oxidation (M), acetyl (protein N-term); variable modifications, iTRAQ8plex (N-term), iTRAQ8plex (K) and iTRAQ8plex (Y); peptide mass tolerance, ± 15 ppm; fragment mass tolerance, 20mmu; peptide confidence, high; quantification experimental bias, normalize on protein median; and peptide FDR < 0.01. The match details of the identified peptides were listed in **Supplementary Dataset S1**. Mass spectrometry proteomics data were deposited in the ProteomeXchange Consortium *via* the PRIDE partner repository with the dataset identifier PXD013784. Student’s *t*-test was employed to assess the statistical significance of the comparison of each protein level between wild type and *mdn1-1*. The significantly affected proteins in *mdn1-1* were identified based on an accumulation fold change (*mdn1-1*/wild type) greater than 1.5 (up-accumulation) or less than 0.667 (down-accumulation) and a *P*-value less than 0.05. GO enrichment analysis was performed as described in our previous study (Wang et al., 2018).

### RNA Extraction and Reverse Transcription-Quantitative PCR (RT-qPCR)

Samples were collected, frozen in liquid nitrogen, and stored at −79°C until use. For RNA isolation, the collected samples were ground to fine powder in liquid nitrogen. Total RNA was isolated using a RNAiso Plus Reagent Kit (Takara, Japan) and further purified with a DNase Kit (Takara, Japan). RT-qPCR was performed according to our previous study (Li et al., 2019). The primers for detecting *SESA3* mRNA were 5’-AAGACAGAGATGCCAGAA-3’ and 5’-CCTCATTTGCTTGCTCAT-3.’ The primer set for detecting *SESA4* mRNA was 5’-ACCAGAGCAAGTCAGGAA-3’ and 5’-TTAGTAGTAAGAAGGGATTGAAGG-3.’ The primer set for detecting *EM1* mRNA was 5’-GGACTCAGTACGATGGAA-3’ and 5’-TTGTTGGTGAACTTTGACT-3.’ The primers for detecting the *MDN1* mRNA were reported in our previous study (Li et al., 2016).

### Germination Assays

Mature seeds of different genotypes to be compared were harvested on the same day. Surface sterilized seeds were plated on the medium described above, stratified at 4°C for 3 d, and then transferred to the growth chamber described above. The percentage of seeds with radicle emergence (germination) was calculated at 1∼5 days after stratification. Three biological replicates were performed, and each contained five technical replicates.

### Image Acquisition and Yield-Related Trait Measurement

The image displaying the dry seed phenotype was acquired *via* stereomicroscopy (OLYMPUS, SZX16). The images displaying the seedling phenotype were acquired with a digital camera (CANON 600D). The seed length and width were measured using the “measuring tool” of the incident software of OLYMPUS SZX16. The hundred-grain weight was measured with an analytical balance (OHAUS, AR224CN).

### Statistical Analysis

The data were statistically analyzed using Student’s *t*-test and one-way ANOVA (Duncan’s multiple range test) in SPSS 17.0 software to investigate the significant differences among groups.

## Results

### *mdn1-1* Exhibits Increased Seed Size

**Figure 1:**
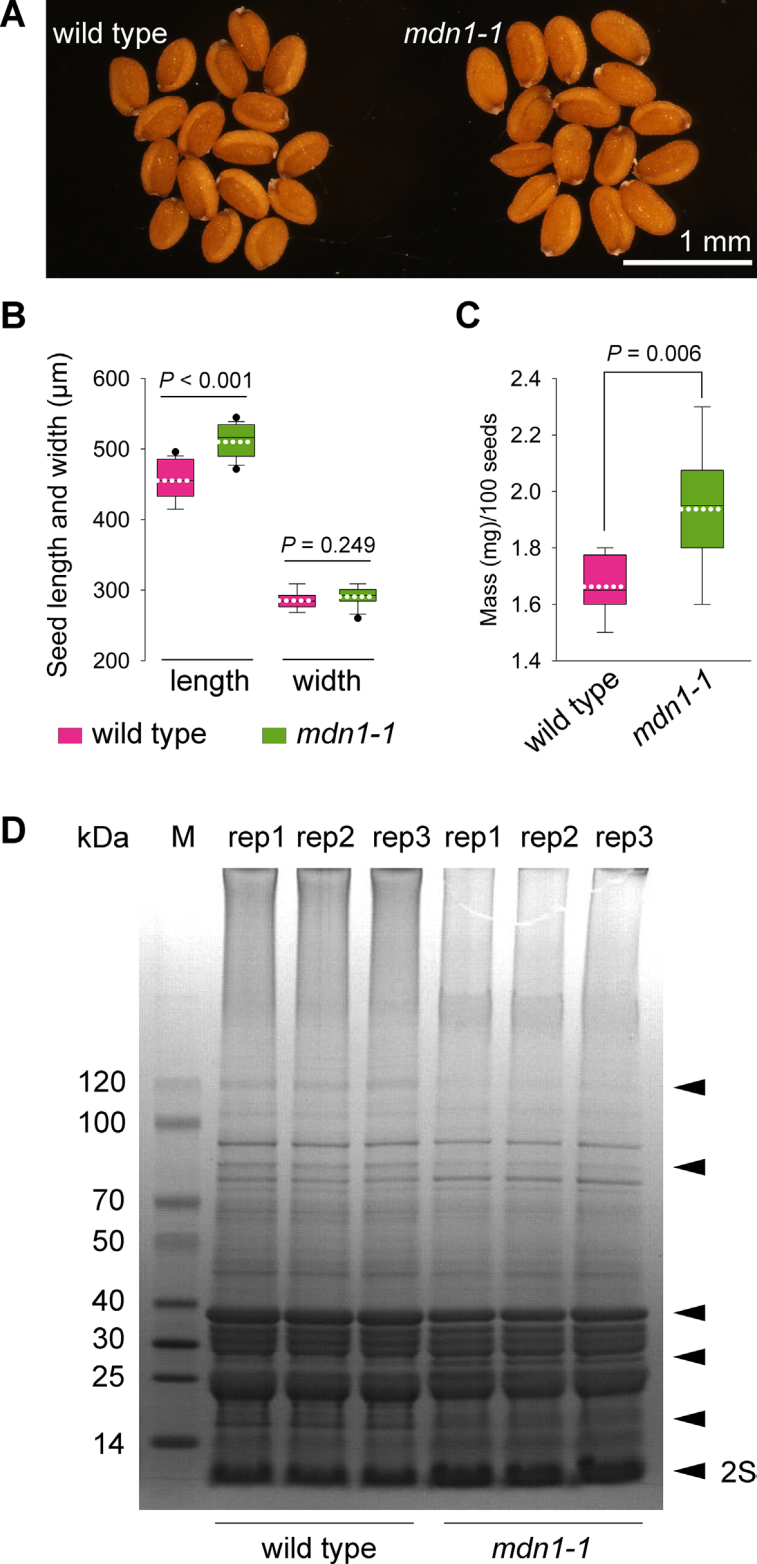
*mdn1-1* displays an increased seed-sized phenotype. **(A)** The dry seed phenotype of wild type and *mdn1-1*. The bar is indicated. **(B)** Comparison of the seed length and width between wild type and *mdn1-1* (n = 17). **(C)** Comparison of the hundred-grain weight between wild type and *mdn1-1* (n = 20). For **(B)** and **(C)**, each box plot shows a median (centerline), the upper and lower quartiles (box limits), maximum and minimum (whiskers), and mean (white dotted line). Student’s *t*-test was applied, and the *P* values are indicated. **(D)** SDS-PAGE analysis of total proteins isolated from dry seeds of wild type and *mdn1-1*. Each lane was loaded with 25 μg protein. Three biological replicates (rep) were performed and indicated. The black arrowheads indicate the affected proteins in *mdn1-1*. 2S, 2S SSPs. M, protein marker.

Previously, we reported that the germination rate of the *mdn1-1* seeds was significantly lower than that of wild type under normal growth conditions (Li et al., 2016). We also found that many of the *mdn1-1* seeds were shriveled and not viable due to impaired embryogenesis (Li et al., 2019). However, the plump seeds of *mdn1-1* appeared to be slightly larger than that of wild type (**Figure 1A**). We further confirmed that *mdn1-1* seed length was significantly longer than that of wild type, while their seed widths were similar (**Figure 1B**). Consistently, the hundred-grain weight of *mdn1-1* was also significantly increased (**Figure 1C**).

*mdn1-2* is a knockout allele of *MDN1* based on a T-DNA insertion, and its homozygote is embryonic lethal (Li et al., 2016; Li et al., 2019). In previous work, we obtained hybrids by crossing heterozygous *mdn1-2* with homozygous *mdn1-1*, and the viable *mdn1-1 mdn1-2* (bi-allelic mutations in *MDN1*) mutant was further isolated from the hybrids (Li et al., 2016). To further determine the effect of MDN1 dysfunction on seeds, we assessed the seed-sized and germination rate phenotypes for the viable progeny (plump seeds) of *mdn1-1 mdn1-2* and found that these results were similar to those for the homozygote of *mdn1-1* (**Supplementary Figure 2**). In addition, through analyzing the relative expression level of *MDN1* during seed maturation using RT-qPCR, we found that its mRNA abundance gradually increased with seed maturation, and the highest level was noted in dry seeds (**Supplementary Figure 3**), although *MDN1* is predominantly expressed in meristematic regions during seedling development (Li et al., 2016). Together, these results suggest that MDN1 plays roles in regulating both seed formation and germination.

Since seed is a protein-rich organ, the above phenotypes and the involvement of MDN1 in ribosome biogenesis led us to test whether the seed proteome was altered by MDN1 dysfunction. Due to homozygosity and viability, *mdn1-1* was employed as the primary material for studying the roles of MDN1. Total proteins were extracted from dry seeds of *mdn1-1* and subjected to SDS-PAGE analysis (**Figure 1D**). The result showed that signals of several bands were clearly changed in *mdn1-1* (**Figure 1D**), suggesting that MDN1 dysfunction affects these protein levels. Notably, the levels of 2S SSPs seemed to be increased in *mdn1-1* (**Figure 1D**), which positively correlated with the increased seed size.

### Identification of MDN1-Dependent Proteome Changes in Seeds

**Figure 2:**
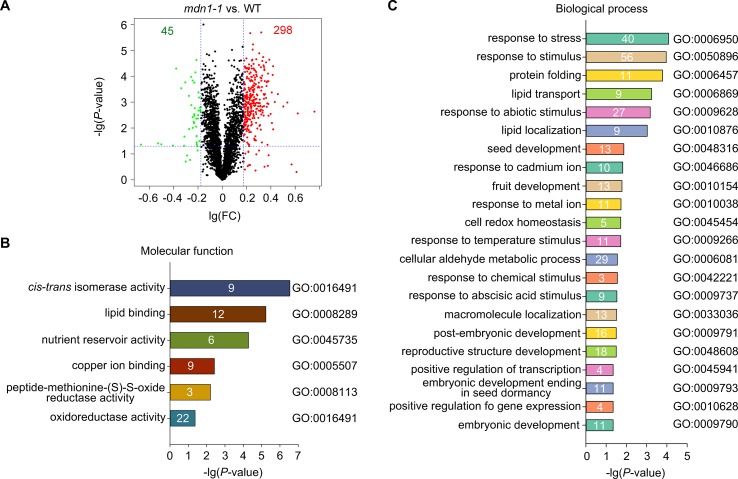
Comparison of seed proteome between wild type and *mdn1-1*. **(A)** A volcano plot showing changes of protein levels in *mdn1-1* compared to those in wild type based on the iTRAQ results. FC, fold change. The red dots indicate the significantly up-DAPs (FC > 1.5, *P* < 0.05), while the green dots indicate the significantly down-DAPs in *mdn1-1* (FC < 0.667, *P* < 0.05). The whole data were listed in **Supplementary Datasets S2** and **S3**. **(B**, **C)** The significantly enriched GO terms (*P* < 0.05) with the DAPs according to the Gene Ontology of molecular function and biological process, respectively. The enriched protein number in each of the GO terms was indicated. The GO terms were sorted based on the *P* values.

To further assess proteome changes in *mdn1-1* seeds, we applied iTRAQ-based LC-MS/MS analysis (Ross et al., 2004). Three biological replicates were performed for increased accuracy. A total of 3,236 proteins were identified from extractions of dry seeds, and 3,217 of these proteins were detected in both wild type and *mdn1-1* (**Figure 2A**, **Supplementary Dataset S2**). The significantly differentially accumulated proteins (DAPs) were identified based on the fold change (FC > 1.5 or < 0.667) in accumulation level and *P*-value (< 0.05) (**Figure 2A**). We identified 282 up- and 40 down-DAPs in *mdn1-1* (**Figure 2A**, **Supplementary Dataset S3**). We further performed Gene Ontology (GO) enrichment analysis on these DAPs. Based on the protein molecular function, many DAPs possessed isomerase activity, lipid-binding activity, and oxidoreductase activity (**Figure 2B**). In terms of the biological processes, the GO terms associated with seed development were enriched with these DAPs, including GO:0048316 (seed development), GO:0009791 (post-embryonic development), and GO:0009790 (embryonic development) (**Figure 2C**). Interestingly, the most enriched GO term was GO:0006950 (response to stress) (**Figure 2C**). Furthermore, GO:0046686 (response to cadmium ion), GO:0009266 (response to temperature stimulus), GO:0042221 (response to chemical stimulus), and GO:0009737 (response to ABA stimulus) were also significantly enriched by the DAPs (**Figure 2C**), suggesting that MDN1 dysfunction might affect the signaling involved in seed perception and response to the external environments or trigger some stress-related signals *in vivo*. As a result of the enrichment of GO:0006457 (protein folding), protein post-translational regulation might be affected in *mdn1-1* (**Figure 2C**). Together, these results reveal that MDN1 plays a role in establishing a normal proteome in seed, which is essential for normal seed development.

### Seed Maturation Proteins Are Over-Accumulated in *mdn1-1* Seeds

**Figure 3:**
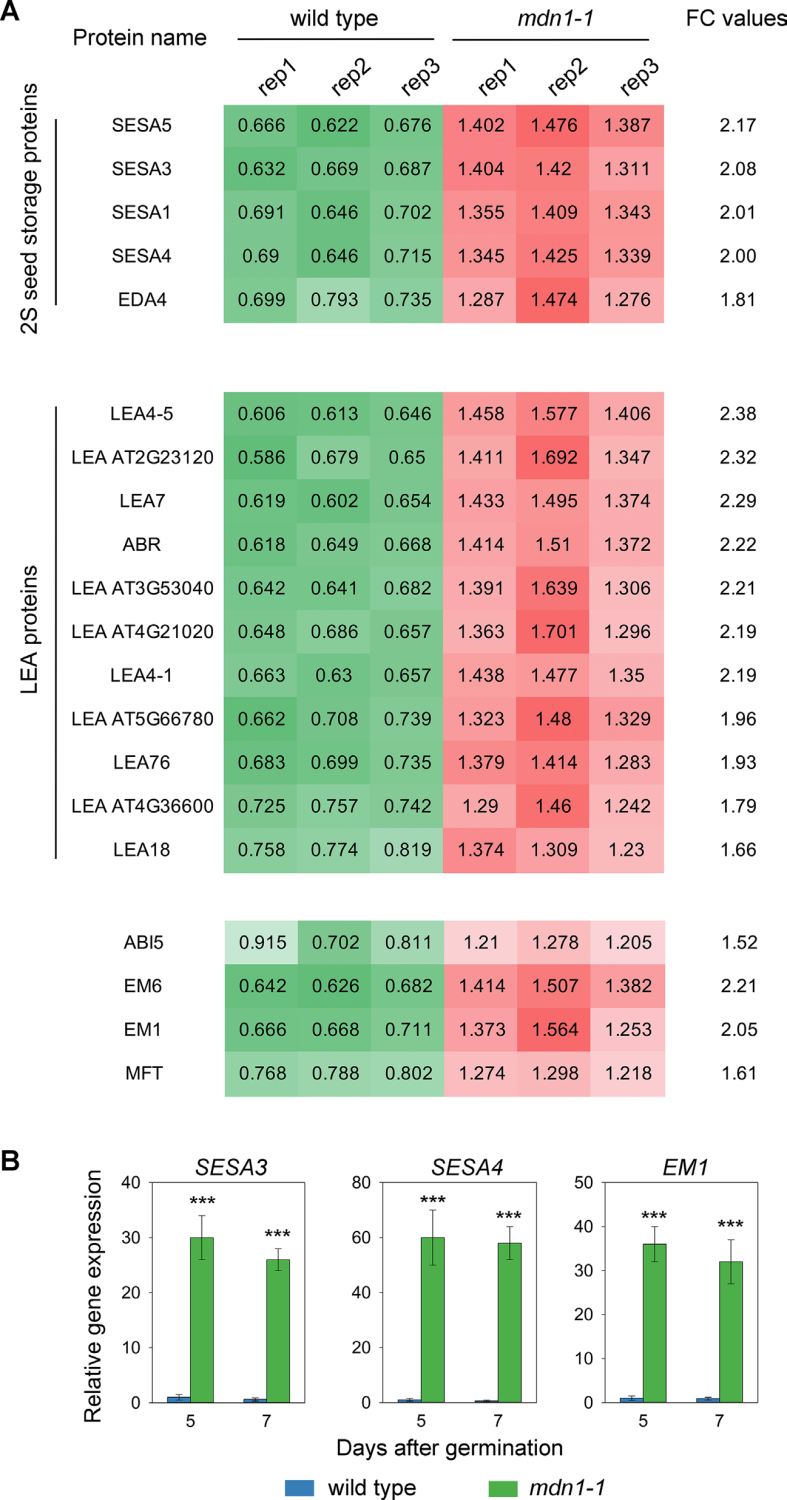
Differences in seed maturation gene expression between wild type and *mdn1-1*. **(A)** The accumulation levels of 2S SSPs, LEA proteins, ABI5, EM1, EM6, and MFT in dry seeds of wild type and *mdn1-1* based on the iTRAQ results. The whole data were listed in **Supplementary Datasets S2** and **S3**. FC, fold change; Rep, replicate; **(B)** Relative expression levels of *SESA3*, *SESA4*, and *EM1* in wild type and *mdn1-1* seedlings grown under normal conditions for 5 and 7 d detected by RT-qPCR. The values were normalized using *ACT2*. Wild-type RNA levels were set to 1. Error bars represent SD (n = 5). Student’s *t*-test was applied (*** indicates *P* < 0.001).

As shown in **Figure 1D**, the 2S SSPs appeared to be over-accumulated in *mdn1-1*. Of the DAPs, we identified five 2S SSPs, including seed storage albumin (SESA) 5, SESA3, SESA1, SESA4, and embryo sac development arrest (EDA) 4, and their levels were increased approximately 1.8- to 2.2-fold in *mdn1-1* compared with wild type (**Figure 3A**). In addition, a 1.7- to 2.4-fold accumulation of 11 LEA proteins was noted in *mdn1-1* seeds (**Figure 3A**). More interestingly, we found that the abundance of ABI5 protein was also increased greater than 1.5-fold in *mdn1-1* (**Figure 3A**). Consistently, the levels of proteins encoded by the ABI5-induced genes, including two LEA proteins (early methionine-labeled 6 [EM6] and EM1) and mother of FT and TFL1 (MFT), were also increased significantly (**Figure 3A**). Given that the above proteins are required for seed maturation, these observations suggest that MDN1 might be involved in this process.

### *mdn1-1* Seedlings Express Seed Maturation Genes

In our previous study, we analyzed MDN1-dependent transcriptome changes in 5-d-old seedlings by RNA sequencing (Li et al., 2016). In this study, we reviewed these data and found that the transcripts of numerous genes involved in seed maturation were overexpressed in *mdn1-1* seedlings. To further confirm these results, we isolated RNAs from wild type and *mdn1-1* seedlings 5 and 7 days after germination and then tested the mRNA levels of *SESA3*, *SESA4*, and *EM1* using RT-qPCR (**Figure 3B**). The results showed that the transcripts of these three genes were minimally detected in wild type (**Figure 3B**). However, in *mdn1-1*, the transcriptional levels of these three genes were dramatically increased (**Figure 3B**). Together with the iTRAQ results, these observations suggest that MDN1 is essential for maintaining the expression homeostasis of the seed maturation genes at both seed and seedling stages.

### *ABI5* Partially Rescues the Low Germination Rate of *mdn1-1*

**Figure 4:**
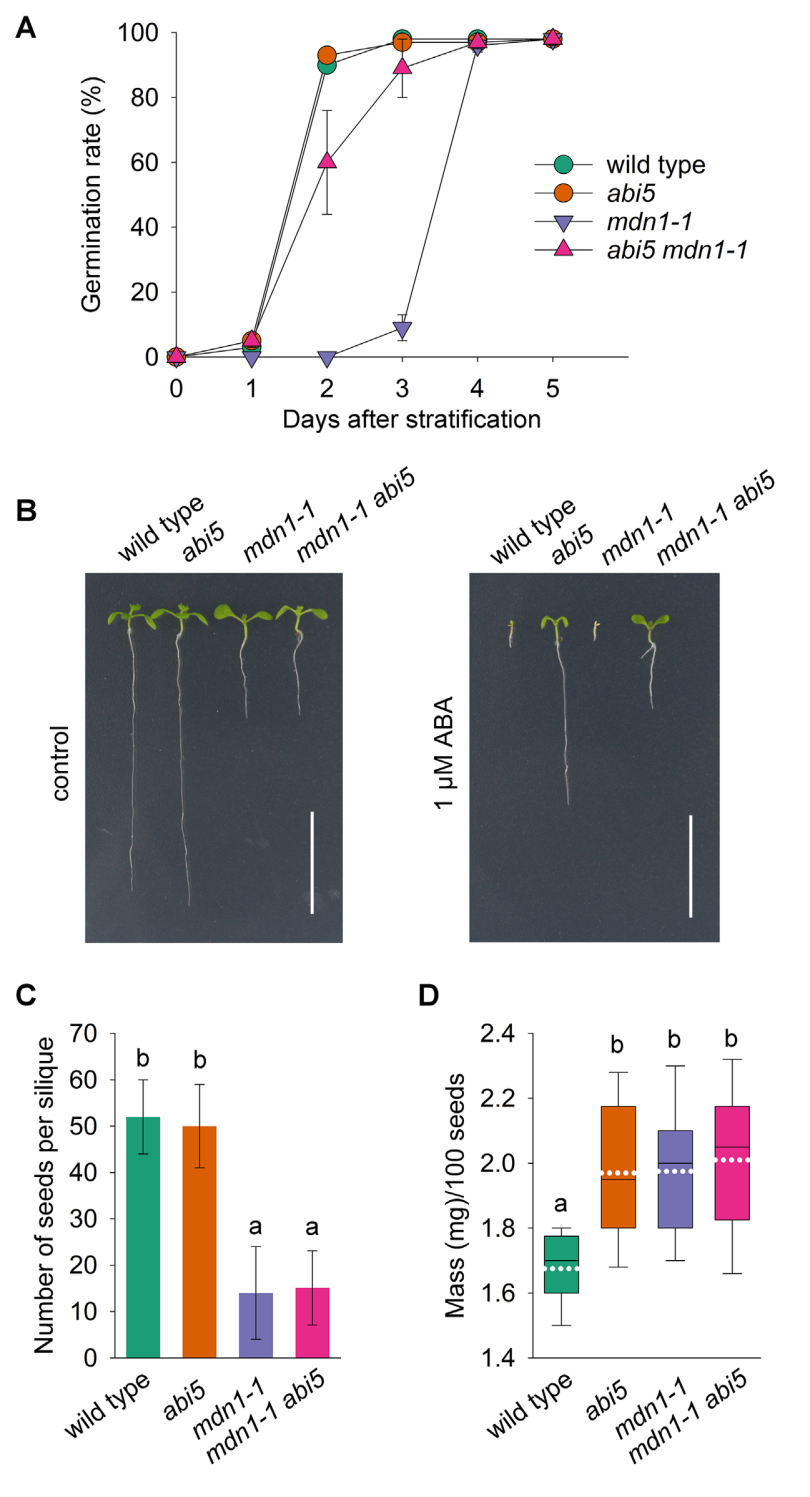
The effect of *abi5* on the phenotypes of *mdn1-1*. **(A)** A time-course analysis of germination rates for the indicated lines grown under normal growth conditions. Each value represents the average germination percentage of 100 seeds with the SD of all replicates. Three biological replicates were performed, and each of which contained five technical replicates. **(B)** Phenotypes of the indicated lines grown under control or 1 μM ABA conditions for 10 days after stratification. Bars = 10 mm. **(C)** The number of plump seeds per silique of the indicated lines. Error bars represent SD (n = 20). **(D)** The hundred-grain weight of the indicated lines. Each box plot shows a median (centerline), the upper and lower quartiles (box limits), maximum and minimum (whiskers), and mean (white dotted line) (n = 20). For **(C)** and **(D)**, one-way ANOVA (Duncan’s multiple range test) was performed, and statistically significant differences are indicated by different lowercase letters (*P* < 0.05).

The increased accumulation of ABI5 and its downstream targets implied that ABI5-mediated signaling was affected in *mdn1-1* seeds. To further investigate whether the impaired homeostasis of ABI5 was linked with the low germination rate of *mdn1-1* seed, we crossed *abi5* with *mdn1-1*. Through detecting the germination rate under normal growth conditions, we found no clear difference between wild type and *abi5* seeds (**Figure 4A**). Notably, the germination rate of *mdn1-1* was significantly increased by the loss of ABI5 function, which was still lower than that of wild type (**Figure 4A**). The result indicates that ABI5 acts genetically downstream of MDN1 during seed germination.

*mdn1-1* exhibits pleiotropic developmental phenotypes (Li et al., 2016). The above results showed that the low germination rate of *mdn1-1* was partially rescued by *abi5*. Unsurprisingly, the loss of ABI5 function also conferred an ABA-insensitive feature on *mdn1-1* during seedling establishment (**Figure 4B**). Nevertheless, *abi5* had no effect on the short-root phenotype of *mdn1-1* under normal and ABA conditions (**Figure 4B**). In addition, we also assessed the yield-related traits of the double mutant *abi5 mdn1-1*. The results showed that both the number of plump seeds per silique and the hundred-grain weight of *abi5 mdn1-1* were similar to *mdn1-1* (**Figures 4C, D**), suggesting that these developmental phenotypes of *mdn1-1* were ABI5-independent.

## Discussion

MDN1 dysfunction in *mdn1-1* leads to the ribosomal stress response given its essential roles in nuclear export of the pre-60S ribosomal particle and maturation of pre-rRNAs (Li et al., 2019). Generally, ribosomal stress is concomitant with multiple developmental defects throughout the plant life cycle, including impaired embryogenesis, pointed and narrow leaves, retarded root growth, altered flowering time, and reduced fertility (Byrne, 2009; Weis et al., 2015a). In addition to *mdn1-1*, the low germination rate phenotype has been reported in other mutants with impaired ribosome biogenesis, such as *apum23-1*, *brx1-2*, and *apum24-2*. *APUM23*, as an *Arabidopsis* Pumilio-encoding gene, is involved in processing and/or degradation of rRNA maturation by-products (Abbasi et al., 2010). Biogenesis of ribosomes in *Xenopus* 1 (BRX1) functions in maintaining homeostasis of a subset of pre-rRNA intermediates, including 33S, 32S, 27SA_2_, and P-A_3_ (Weis et al., 2015b). *APUM24* is also an *Arabidopsis* Pumilio-encoding gene, which is vital for the removal of the internal transcribed specer2 (ITS2) of the pre-rRNA (Maekawa et al., 2018). Therefore, we conclude that the low germination rate in *mdn1-1* might be a ribosomal stress response.

MDN1 is required for embryo development (Li et al., 2019). Moreover, *mdn1-1* displays a large-seed phenotype. Nevertheless, the per silique yield is not increased in *mdn1-1*, because its silique is significantly shorter than that of wild type and contains many unviable shriveled seeds. In addition, given that the seed size phenotype is not mentioned in other mutants with impaired ribosome biogenesis, we remain unsure whether the large-seed phenotype of *mdn1-1* is a MDN1-specific effect or ribosomal stress involved.

Through a proteomic comparison, we found that the abundance of five 2S SSPs was significantly increased in *mdn1-1*, which might contribute to the mutant large-seed phenotype. However, 12S SSP levels were not sensitive to MDN1 dysfunction. The uncoupled expression patterns of 2S and 12S also suggest that they may have different regulation pathways during seed filling. Compared with the limited knowledge on 12S SSP gene regulation, 2S gene regulation has been well studied. Several transcription factors have been identified as the regulators of 2S SSP genes, including ABI3, FUSCA 3 (FUS3), LEAFY COTYLEDON 2 (LEC2), bZIP10, bZIP25, and bZIP53. ABI3, FUS3, and LEC2 belong to the B3-domain family and induce 2S gene transcription through binding the RY-motif (Kroj et al., 2003). Synergistically, bZIP53 interacts with bZIP10 or bZIP25 to form a heterodimer, which targets the G-box motif for the 2S gene activation (Alonso et al., 2009). Given that the transcription of these genes is dramatically downregulated at the late stage of seed maturation, their proteins are minimally detected in dry seeds.

In addition to SSPs, many LEAs were also up-accumulated in dry seeds of *mdn1-1*. *LEA* gene expression in seeds is tightly associated with ABA signaling given that promoters of approximately 82% of all *LEA* genes in *Arabidopsis* contain the ABA-responsive element (ABRE) motif (Hundertmark and Hincha, 2008). Furthermore, ABI5, a key mediator of ABA signaling, was also over-accumulated in *mdn1-1*. These results suggest that ABA signaling is more active in *mdn1-1* than in wild type during seed maturation.

More interestingly, some of the *SSP* and *LEA* genes were highly expressed in *mdn1-1* seedlings, suggesting that the repression pathway of these genes in seedling is affected by MDN1 dysfunction. Many factors are involved in repressing seed maturation genes in seedlings. For instance, the Brahma (BRM)-containing ATPase complex mediates the repression of SSPs in leaves through remodeling the chromatin structures of their gene promoters (Tang et al., 2008). High-level expression of sugar-inducible gene 2 (HSI2) and HSI2-like 1 (HSL1) are B3-domain factors and play a key role in seedling development through inhibiting the expression of seed maturation genes (Tsukagoshi et al., 2007; Zhou et al., 2013). The genetic relationship between these factors and MDN1 in repressing seed maturation genes during seedling growth need to be studied further, which might be conductive to clearly elucidating the repression mechanism.

We further found that the loss of ABI5 function partially rescued the low germination rate of *mdn1-1*, suggesting that ABI5 is responsible for preventing seed germination when the MDN1 function is impaired. Since the function of ABI5 is phosphorylation-dependent (Piskurewicz et al., 2008), MDN1 dysfunction might not only increase ABI5 protein levels, but also affect its post-translational modification. In addition, as both a downstream responding factor and an upstream repressor of ABI5 (Xi et al., 2010), MFT was also up-accumulated in *mdn1-1* seeds. As a result, the steady state of the regulatory loop formed with ABI5, and MFT might be influenced as well by MDN1 dysfunction.

Based on our previous and present results, we demonstrate that MDN1 is vital for normal seed development through not only regulating early embryo development but also establishing a normal seed proteome during the maturation stage. Given the molecular function of MDN1 in ribosome biogenesis, our observations further imply that a link might exist between ribosome biogenesis and ABA and GA signaling during seed maturation and germination, which remains a fascinating subject for future study.

## Data Availability

All datasets for this study are included in the manuscript and the supplementary files.

## Author Contributions

P-CL and X-JW designed the research. P-CL, J-JM, and X-MZ performed the research. P-CL, J-JM, X-MZ, G-HL, C-ZZ, HX, S-JF, and X-JW analyzed the data. P-CL wrote the paper.

## Funding

This work was supported by the National Natural Science Foundation (Peng-Cheng Li, 31500257), the Agricultural scientific and technological innovation project of Shandong Academy of Agricultural Sciences (CXGC2018E13).

## Conflict of Interest Statement

The authors declare that the research was conducted in the absence of any commercial or financial relationships that could be construed as a potential conflict of interest.
