# Supplementary figures and images for: Arabidopsis MDN1 Is Involved in the Establishment of a Normal Seed Proteome and Seed Germination
Source: Front Plant Sci. 2019 Sep 10;10:1118. doi: 10.3389/fpls.2019.01118 (PMC6746975; doi:10.3389/fpls.2019.01118)

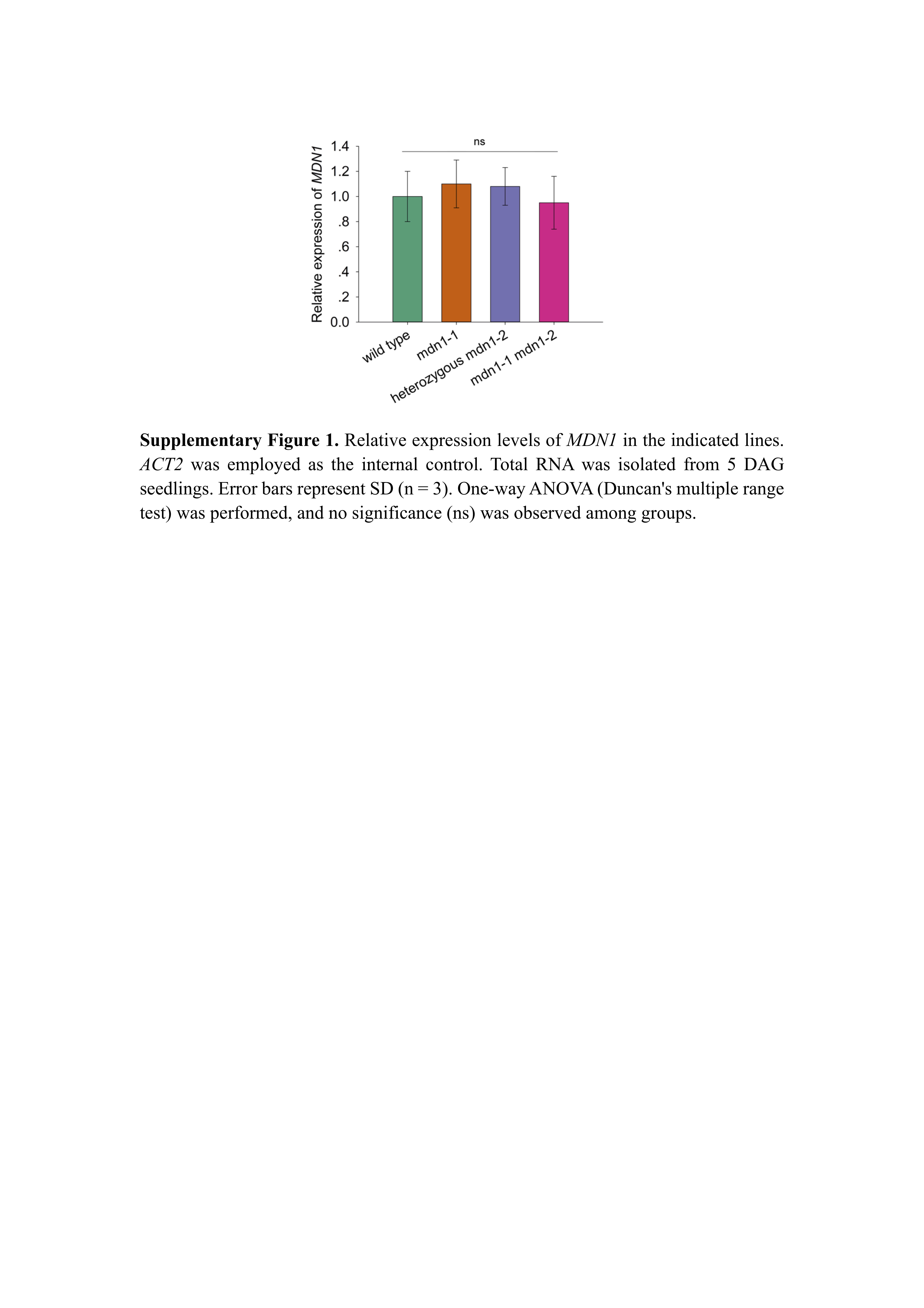

Supplement: Supplementary file 1 [file Image_1.jpg]

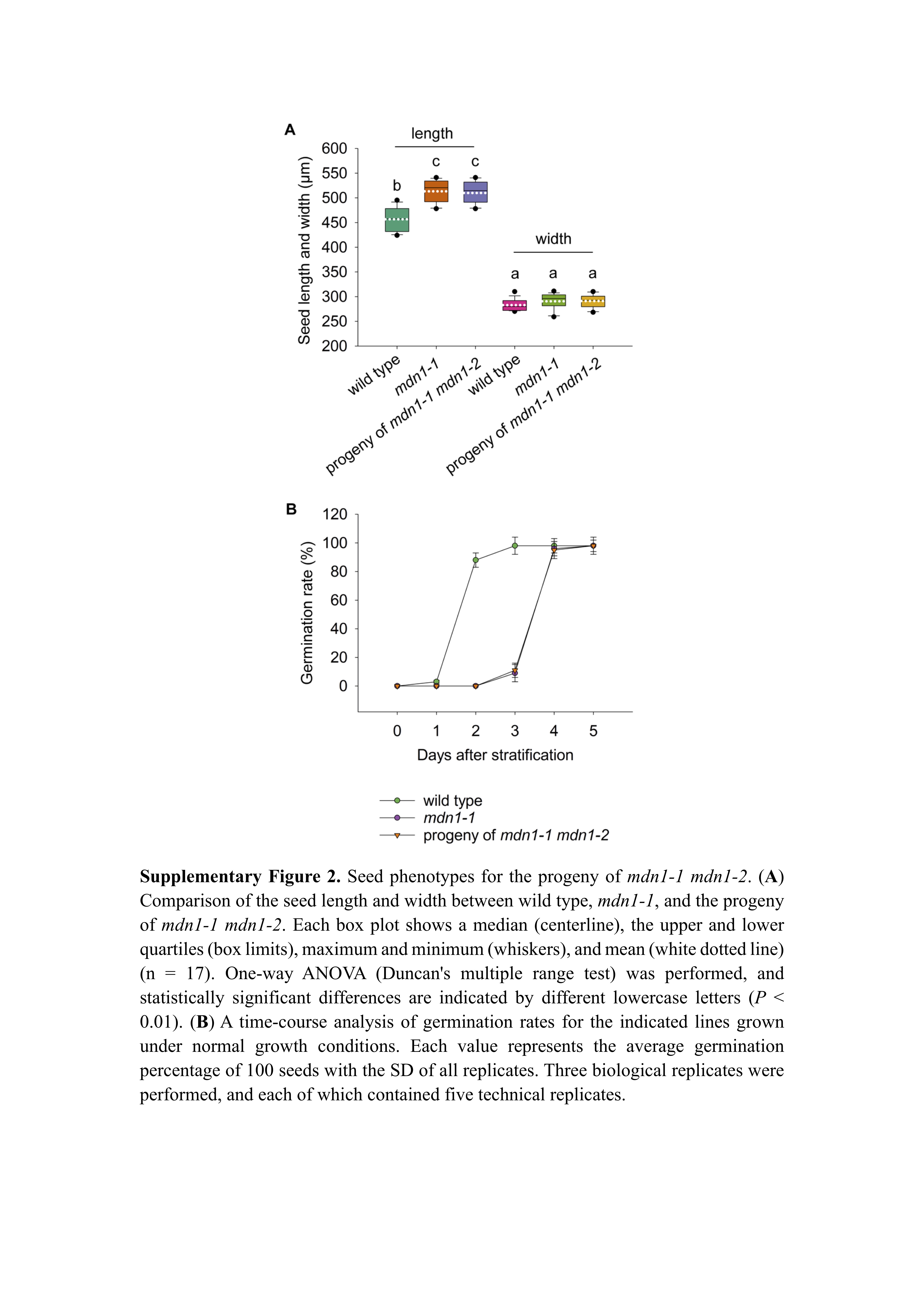

Supplement: Supplementary file 2 [file Image_2.jpeg]

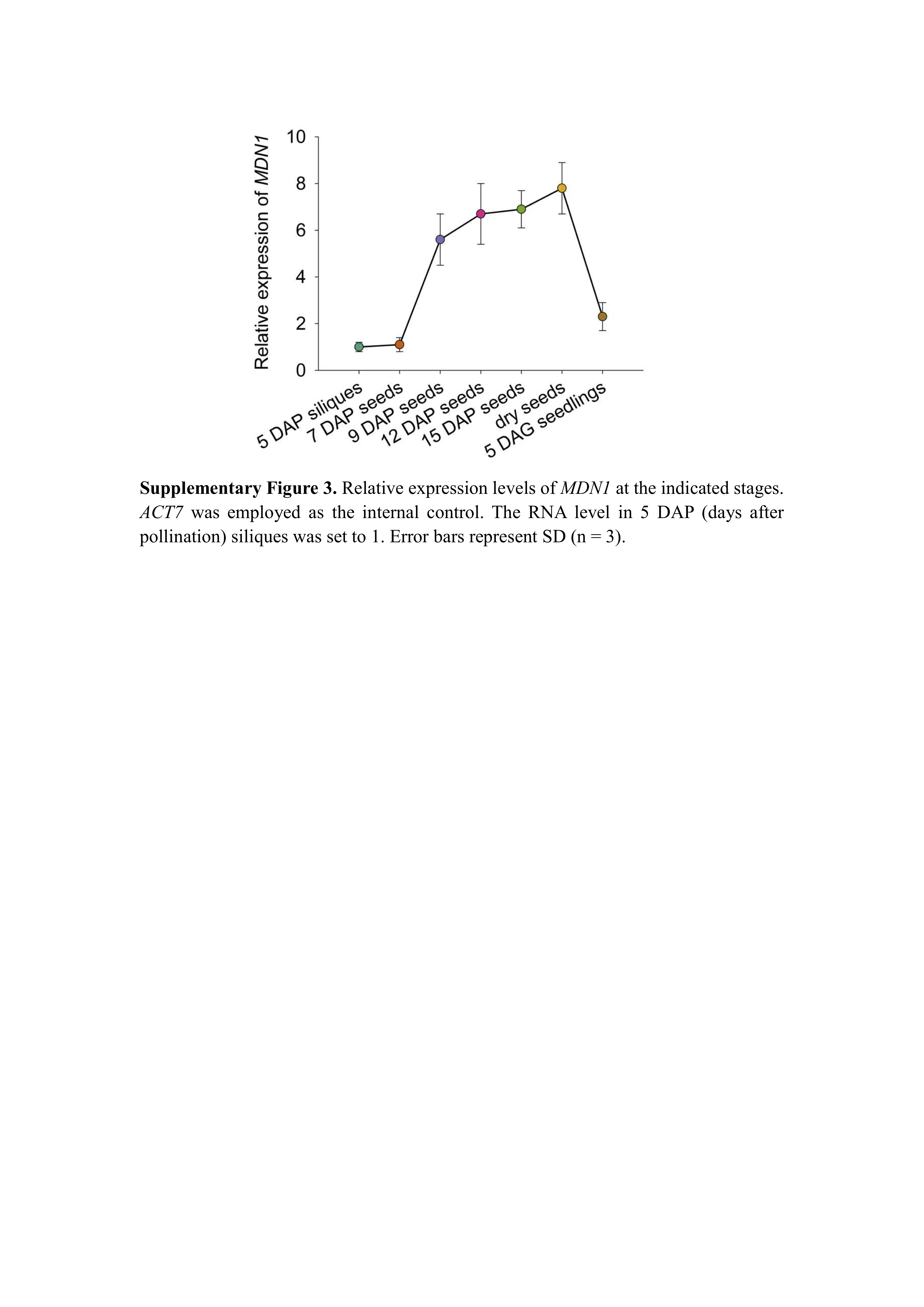

Supplement: Supplementary file 3 [file Image_3.jpeg]
